# Biomimetic Hydrogels in the Study of Cancer Mechanobiology: Overview, Biomedical Applications, and Future Perspectives

**DOI:** 10.3390/gels8080496

**Published:** 2022-08-10

**Authors:** Ayse Z. Sahan, Murat Baday, Chirag B. Patel

**Affiliations:** 1 Biomedical Sciences Graduate Program, Department of Pharmacology, School of Medicine, University California at San Diego, 9500 Gilman Drive, San Diego, CA 92093, USA; 2 Department of Neurology and Neurological Sciences, School of Medicine, Stanford University, Stanford, CA 94305, USA; 3 Precision Health and Integrated Diagnostics Center, School of Medicine, Stanford University, Stanford, CA 94305, USA; 4 Department of Neuro-Oncology, The University of Texas MD Anderson Cancer Center, Houston, TX 77030, USA; 5 Neuroscience Graduate Program, The University of Texas MD Anderson Cancer Center UTHealth Graduate School of Biomedical Sciences (GSBS), Houston, TX 77030, USA; 6 Cancer Biology Program, The University of Texas MD Anderson Cancer Center UTHealth Graduate School of Biomedical Sciences (GSBS), Houston, TX 77030, USA

**Keywords:** cancer, glioblastoma, hydrogel, mechanobiology, mechanoreceptor, mechanotransduction

## Abstract

Hydrogels are biocompatible polymers that are tunable to the system under study, allowing them to be widely used in medicine, bioprinting, tissue engineering, and biomechanics. Hydrogels are used to mimic the three-dimensional microenvironment of tissues, which is essential to understanding cell–cell interactions and intracellular signaling pathways (e.g., proliferation, apoptosis, growth, and survival). Emerging evidence suggests that the malignant properties of cancer cells depend on mechanical cues that arise from changes in their microenvironment. These mechanobiological cues include stiffness, shear stress, and pressure, and have an impact on cancer proliferation and invasion. The hydrogels can be tuned to simulate these mechanobiological tissue properties. Although interest in and research on the biomedical applications of hydrogels has increased in the past 25 years, there is still much to learn about the development of biomimetic hydrogels and their potential applications in biomedical and clinical settings. This review highlights the application of hydrogels in developing pre-clinical cancer models and their potential for translation to human disease with a focus on reviewing the utility of such models in studying glioblastoma progression.

## 1. Cellular Microenvironment

The cellular microenvironment is characterized by a mixture of extracellular matrix proteins, soluble signaling factors, neighboring cells, and the physical properties of the niche that affect cell behavior through direct or indirect biomechanical and biochemical signals [1,2]. Properties of the microenvironment, such as stiffness and composition, have been shown to direct cell physiology and lineage [3,4]. Such findings have inspired research to define the cellular microenvironment and its links to cellular behaviors using in vitro tissue models that can mimic biomechanical conditions [5,6]. Here, we will discuss the cellular niche, namely, the biological and mechanical properties of the extracellular matrix (ECM), how cells sense these properties, and the dysregulation of cell-ECM interactions in various disease states. How these factors, which are involved in crosstalk with cells, contribute to cellular activities and overall health, will also be presented through a review of the various research publications on the topic. This review will provide an in-depth overview of what is known and what is unknown about the biomechanics involved in cell-microenvironment interactions, how the use of biomimetic hydrogel models can fill these gaps in the knowledge, and the utility of biomimetic hydrogels in biomedical applications.

### 1.1. Extracellular Matrix

**Figure 1:**
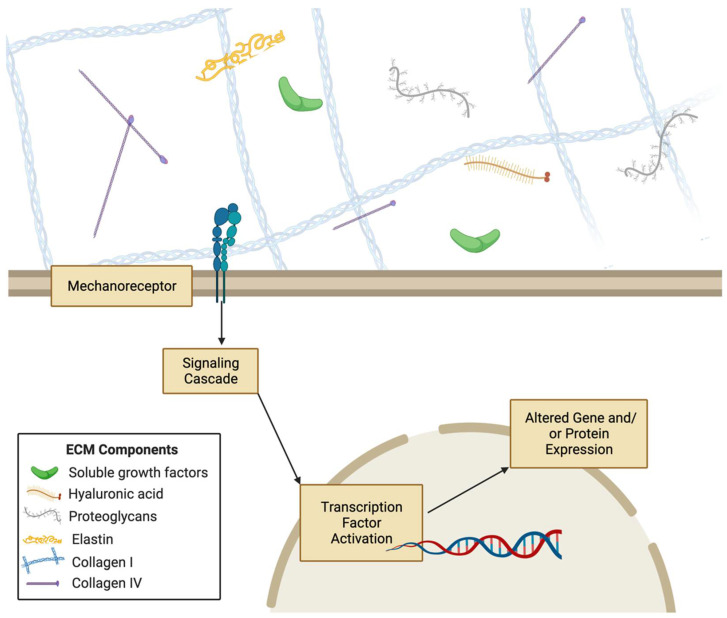
Extracellular matrix (ECM) and cellular mechanotransduction. ECM components such as collagens can alter mechanical properties to induce changes in cellular signaling and gene or protein expression via mechanoreceptors and mechanotransduction proteins. Legend on the bottom left shows which ECM component is represented by each symbol. (Created with BioRender.com, accessed on 29 May 2022).

Tissues may be described as having two components: cellular and non-cellular. The extracellular matrix is the non-cellular component and is composed of proteins, polysaccharides, growth factors, signaling molecules, proteases, and water [7] (Figure 1). These components are distributed heterogeneously rather than homogenously, resulting in unique niche microenvironments for each cell as well as tissue-specific mechanical, physical, and biochemical properties [7]. These play a large role in regulating and mediating cell behaviors. While the ECM is a mixture of many components, a large portion of it is composed of proteins. These include proteoglycans such as hyaluronan, and fibrous proteins such as collagens, elastins, fibronectins, and laminins [8]. These proteins function to anchor cells to the ECM via focal adhesions and aid propagating signals between cells [8]. ECM composition also affects physical properties such as elasticity, stiffness, porosity, static architecture, and dynamic deformations of the matrix [9]. For instance, based on their concentration, assembly, and crosslinking densities, the structural collagens and elastins of the ECM significantly alter its mechanical properties such as composite strength, elasticity, and mechanical resistance [10,11]. ECM properties are not static; they undergo dynamic changes as the ECM is continuously being remodeled through protein degradation, deposition, or modifications that can be self-contained or caused by cellular activity [10]. In cases such as tissue repair, the activity of growth factors and cytokines in the ECM cause matrix metalloproteinases to activate for ECM remodeling [12]. Wound healing and tissue remodeling processes activate growth factors through mechanical and biochemical stimuli to change ECM composition [13]. There are also specialized forms of ECMs that have proven to be important regulators of tissue and cell behavior. For instance, basement membrane is important structurally and functionally for blood vessels because of its involvement in angiogenesis [12]. The ECM has also been shown to mediate or regulate stem cell fate, cell proliferation, cell differentiation, cell migration, and tissue regeneration [11,14,15,16].

#### 1.1.1. Cell-ECM Interactions

The cell and its ECM are involved in a dynamic reciprocity, through which cues from the ECM and cellular activities are in crosstalk to maintain a healthy state [17]. Signaling processes are one of the ways that the cell-ECM interactions are facilitated. Cell-matrix adhesion sites, or focal contacts, enable communication between cells and the ECM through physical connections of cellular integrins and cadherins to ligands in the ECM [15,16]. Focal contacts are important to cellular processes that require physical attachment to the ECM, such as migration and angiogenesis [18]. Engagement of integrins and cadherins to certain ligands can activate signaling pathways such as that of the Rho family of GTPases to stimulate structural changes in the cell or induce other processes, therefore serving as an important step in biochemical cell-ECM interactions [6,16].

Proteoglycans present in the ECM have functions such as inducing aggregation and participating in ECM structure by adhering to structural proteins [19]. Some proteoglycans reside on the surfaces of epithelial cells, where they act similar to cell-adhesion molecules and bind collagens and fibronectin to anchor cells to the ECM [20,21]. In addition to these vital functions, proteoglycans are active co-receptors that mediate cellular signaling by binding soluble ligands in the ECM and encouraging the formation of receptor complexes on cell surfaces [22,23]. Proteoglycan co-receptors are vital to various developmental processes, and the loss of co-receptor function or mutation has been implicated in diseases such as cancer and ischemic heart disease [23]. Therefore, the ECM also plays a crucial role in mediating cell–cell communication, which will be discussed more thoroughly in Section 1.2 of this review.

##### Mechanobiology of the Cellular Microenvironment

The ECM is involved in mechanical crosstalk with cells [24], which relies on mechanotransduction proteins that help to regulate intracellular tensile response to mechanical forces from the ECM [25]. Mechanical stimuli that cells may receive include shear stress, membrane tension, force, strain, stiffness, and drag force [25]. These stimuli are listed in Table 1 with the mechanotransduction proteins identified to be involved in recognizing the stimuli and eliciting the response in cells. While the extracellular matrix of many cancers, including colon, breast, and prostate cancer, is stiffer than that of healthy tissues [26,27], cell deformability or reduced stiffness has been correlated to increased metastatic potential and invasiveness in cancer. In a study of ovarian cancer cells of varying invasiveness, Xu et al. found that the more-invasive ovarian cancer cell line was more deformable compared to the less-invasive cell line [28]. In another study, Hayashi and Iwata confirmed that cancer cells are softer (i.e., lower Young’s modulus) than normal cells using atomic force microscopy [29]. Another study reported that cisplatin treatment caused decreased stiffness and invasiveness of prostate cancer cells [30], suggesting that various cell lines may have varied mechanical properties in the cancer state. Further controversy exists within the field of glioblastoma biomechanics. Gliomas are highly variable; therefore, measurements of tumor stiffness may vary depending on location of measurement [31]. Although some GBM tissues were stiffer than healthy reference tissues, GBM tissues, on average, were less stiff than healthy tissues [31,32,33]. The controversy regarding GBM tumor stiffness could by fueled by differences in methods of measurement, as there is currently no standard practice in the field [34,35,36].

**Figure 2:**
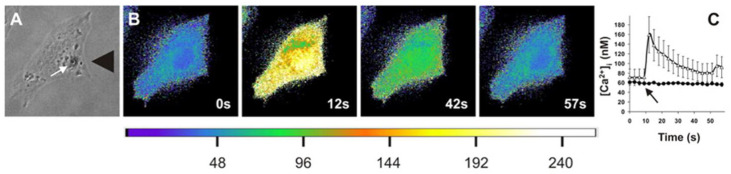
Application of extracellular stress leads to increased intracellular calcium concentrations. Matthews et al. applied high levels of stress to cells (**A**) and found that when imaged via Fura-2AM ratio imaging, it led to a transient increase in calcium concentrations, as shown in pseudocolor images ranging from blue to yellow (**B**) that is quantified (**C**) as a function of time for control and gadolinium chloride-treated cells. (Figure reprinted/adapted with permission from Ref. [61]. Copyright 2006, National Academy of Sciences.

Mechanical stimuli coming from the extracellular environment can be processed by cells through mechanotransduction pathways. The proteins of these pathways translate mechanical cues to induce biochemical and genetic responses. For instance, integrins and focal adhesion proteins are mechanotransduction proteins that communicate mechanical forces to the cell cytoskeleton, and there have been some studies that show force-dependent integrin activation [37,38,39]. Yes-associated protein (YAP) and other transcription factors have been shown to translocate to the nucleus in stiffer substrates [40]. Similarly, E-cadherin is a mechanotranducer of shear stress [41,42,43]. Many mechanotransduction proteins have been studied in the context of specific cell types, including platelet endothelial cell adhesion molecule-1 (PECAM-1) in skeletal muscle cells, G protein-coupled receptors in endothelial cells, and vascular endothelial growth factor receptor 2 (VEGFR2) in chondrocytes [44,45,46,47]. Cell surface receptors also transduce mechanical signals to cells upon recognition of a ligand that sustained force from the ECM, which can then cause conformational changes in the mechanoreceptor to activate a protein signaling pathway to alter cellular processes [48]. Such membrane proteins are not the sole propagators of mechanical force; studies have shown that there are mechanotransduction systems in cells that enable the progression of force through a long distance [49]. Src and Rac1 have been shown to be activated at distances from 30-60 μm from the original area the force was applied to, via cytoskeletal mediation of the force [49,50]. Mechanical signals can also be transmitted throughout the cytoskeleton to the nucleus via proteins such as linker of nucleo- and cyto-skeleton (LINC) complex 9 to change chromatin structure and cause nuclear stiffening [49,51]. Mechanotransduction systems translate the numerous mechanical cues from the ECM into biochemical signals interpreted by the cell that lead to signaling cascades that control transcription, proliferation, migration, and many other cellular processes [52,53,54,55] (Figure 2). For instance, smooth muscle cells can migrate along gradients of substrate stiffness through durotaxis [56]. ECM biomechanics is not only vital for cellular processes, but also regulates tissue and organ-level processes such as tissue differentiation, morphogenesis, and development [57,58,59,60]. Therefore, elucidating the interplay of ECM biomechanical and biochemical signals with mechanotransduction proteins and pathways is critical to understanding diverse aspects of cellular and tissue health.

**Table 1:** Mechanotransducers of various mechanical properties and human cellular responses.

Functional Category	Mechano-Transducers	Mechanical Signal	Examples of Cellular Responses
Cell Mechanical and Physical Properties	Integrins	Force	RhoA activation leading to increased cell stiffness [62,63]
	Focal Adhesions	Force	Actin polymerization [55]
	Yes-associated protein (YAP)	Force	Oligodendrocyte morphology and maturation [40]
	Titin	Force	Implicated in development of mechanical unloading-induced diaphragm weakness [64]
	Stress Fibers (actin filaments, myosin II, etc.)	Force	Transmit tension to other proteins, regulate assembly of filaments [65]
	Vinculin	Force	Transmit tensile force [66]
	Myosin II	Force	Increased cortical tension and cell membrane fusion promotion [67]
	Vasodilator stimulated phosphoprotein (VASP), zyxin, and Testin LIM domain protein (TES)	Force	Regulate junction dynamics [68]
	Neurogenic locus notch homolog protein 1 (NOTCH1)	Shear Stress	Altered cell morphology [69]
	Piezo1	Force	Vascular structure [70]
	Lamin A	Rigidity	Nuclear mechanics [71,72]
	Integrins	Force	Tyrosine Phosphorylation, MAPK signaling [15]
Alters Signaling Pathways	Focal Adhesions	Force	Integrin convergence [73]
	Fibronectin	Force	Altered integrin binding [74]
	T-cell receptor (TCR)	Force	T-cell calcium and IL-2 secretion [75]
	Talin	Force	Recruitment of vinculin to focal adhesion complexes [76]
	Piezo2	Force	Serotonin release [77]
	Vinculin	Force	Enhanced PI3K activation [78]
	p130Cas	Force	Activation of Cas signaling pathway [79]
	Syndecan-1	Force	Activation of pro-inflammatory and growth-stimulating pathways [80]
	Transient Receptor Potential Cation Channel Subfamily V Member 4 (TRPV4)	Force	Reorientation and flow-mediated nitric oxide production [81]
	Ion Channels	Force	Cell signaling [82]
	von Willebrand factor—glycoprotein Ib complex (VWF-GPIb)	Shear Stress	Enhanced calcium triggering in platelets and T cells [83]
	Platelet endothelial cell adhesion molecule-1 (PECAM-1)	Shear Stress	Tyrosine kinase Src and PI3K signaling activated [84]
	G-protein coupled receptor 68 (GPR68)	Shear Stress	Component in signaling for cardiovascular pathophysiology [85]
	β-catenin	Shear Stress	Activated expression of FOXC2 transcription factor [86]
	Caveolin-1 and β1 Integrin	Stiffness	FA assembly and turnover [62]
	rho-associated, coiled-coil-containing protein kinase (ROCK) 1 and 2	Stiffness	Regulation of RhoA signaling pathways [87]
	YAP	Stiffness	Altered translocation depending on surrounding stiffness [88]
	Piezo1	Force	Ion Permeation and selection [89]
	C-X-C motif chemokine receptor (CXCR1/2)	Shear Stress	Mediates laminar shear-stress-induced endothelial cell migration [90]
	Transforming growth factor beta 1 (TGFβ1)	Shear Stress	Collagenase-dependent fibroblast migration [91]
Migration	RhoA	Force	Collective cell migration [92]
	Vinculin and metavinculin	Force	Regulation of cell adhesion and motility [66]
	NOTCH1	Shear Stress	Decreased proliferation [69]
	Caveolin 1	Rigidity	Decreased proliferation [93]
Cancer	YAP1	Shear Stress Stiffness	Cancer cell motility [54] Nuclear localization of YAP1 [94]
	TGFβ1	Shear Stress	Human melanoma cell tumor invasiveness [91]
	PI3K/Akt pathway	Stiffness	Overexpression of VEGF in hepatocarcinoma cells [63]
	TRPV4 ion channel	Stiffness	Tumor vascularization through down-regulation of Rho kinase activity [95]
	microRNAs	Stiffness	Altered expression in different stiffness conditions [96]
	Twist1	Stiffness	Induction of EMT and tumor metastasis [97]
	Myocardin related transcription factor A (MRTF-A)	Stiffness	Regulates miRNAs involved in myogenic differentiation [88]
Differentiation	Focal Adhesions	Force	Osteogenic differentiation [98] Myofibroblastic differentiation [99]
	Transient Receptor Potential Cation Channel Subfamily M Member 7 (TRPM7)	Shear Stress	Osteogenic differentiation of mesenchymal stromal cells [100]

#### 1.1.2. Cell-ECM Interactions in Cancer

Problems in mechanotransduction can result from changes in ECM mechanical properties and defects in proteins involved in mechano-sensitivity [101]. Since mechanotransduction is essential for modulating cellular homeostasis, its failure is linked to metastasis and cancer progression [96]. For instance, there are many proteins implicated in mechanotransduction in glioblastoma (GBM). Talin1 inhibition has been observed to decrease cell spreading and limit cell stiffness changes of glioma cells in response to ECM stiffness, proving its role as a mechanosensory [76]. Non-muscle myosin II depletion reduced the effect of matrix confinement on GBM cell motility [102]. Constitutive activation of RhoA GTPase caused lower sensitivity to matrix stiffness of GBM cells in toxicity assays [55]. Increased matrix stiffness was correlated to Hras, RhoA, and rho-associated, coiled-coil-containing *protein* kinase 1 (ROCK1) upregulation, which are mechanosensor proteins that are implicated in migration and proliferation in cancers in general [61,87]. Integrins are also particularly important in mechanotransduction by relaying signals from the ECM to the cell actin cytoskeleton and are essential to cell migration and cell-matrix adhesion in cancer [103].

Identifying proteins involved in mechanotransduction and their roles in cancer progression can be an essential part of developing therapeutic strategies to hinder cancer progression and malignancy. Certain studies have shown the potential effects of targeting mechanotransduction proteins on cancer cells. Knock-down of CD44 led to decreased structural microtubule, vimentin, and glial fibrillary acidic protein expression and decreased migration and cell stiffness [104]. Targeted inhibition of integrins in the tumor microenvironment has been shown to reduce angiogenesis and inhibit tumor growth [103]. While these studies show partial inhibition of mechanical sensitivity as decreasing invasive properties of tumor cells, other studies have shown that tumor initiating cells are mostly insensitive to mechanical cues from the ECM and that mechanically-insensitive cells have increased motility and invasiveness in vitro [28,105,106,107,108,109]. A complex approach is needed to target mechanical sensitivity in cells through mechanotransduction-based therapeutics for it to become a promising mode of cancer treatment. Therefore, extensive research in mechanotransduction and cell-ECM crosstalk is essential.

### 1.2. Neighboring Cells and Secreted Factors

In addition to the ECM components, cells are surrounded by heterogeneous populations of neighboring cells that are unique to the tissue and sub-location within the tissue [110]. How cells communicate and influence one another is crucial to maintaining homeostasis and coordinating processes that require several cells, such as tissue formation and regeneration.

Cells can interact through secreted signals that are recognized by membrane-bound receptors through either paracrine signaling, between cells, or autocrine signaling, which is from one cell to itself. In fact, cancer cells are often able to “override” signals from neighboring cells through autocrine pro-survival and proliferation signals. It is crucial to better understand the specific interactions between cells that promote healthy conditions or lead to disease states. For instance, Zervantonakis et al. found that fibroblasts in the tumor microenvironment of HER2 positive breast-cancer cells reduced drug sensitivity through paracrine signaling that activates mechanistic target of rapamycin (mTOR, anti-autophagic) and anti-apoptotic signals [111]. In addition to elucidating mechanisms of tumor resistance, cell–cell interaction dynamics can provide insights to developing self-assembled multicellular structures in vitro. Mueller et al. demonstrated that by utilizing pulsed light activation to control engineered photo-switchable cell-cell interactions, they were able to control the spatial organization of multicellular structures without a scaffold [112]. This highlights the importance of maintaining cell interactions in efforts to mimic physiological conditions in vitro.

Cells are also involved in communications through physical contacts with one another. This is another way that mechanical stimuli may play a role in influencing cellular processes. Physical contacts are also involved in collective migration, which has been exhibited by metastatic and invasive cancer cells [113]. The cytoskeletal tension at cell-cell contacts can serve as a significant regulator of mechanotransduction pathways, and there is a wide field of study on the mechano-sensing implications of cell-cell contacts such as focal adhesions and adherens [114]. One method in particular, physically interacting cell sequencing (PIC-seq), is a novel sequencing approach that combines cell sorting, RNA-sequencing, and computational modeling to describe complex cellular interactions in different contexts [115]. However, the heterogeneity and complexity of the interactions between cells and with the ECM need to be better recapitulated in vitro to provide a more accurate understanding of cellular communication networks.

### 1.3. Hydrogels as In Vitro Models of the Cellular Microenvironment

Three-dimensional hydrogel models of the cellular microenvironment have gained interest in recent years for their improved mimicry of in vivo conditions as opposed to two-dimensional cell cultures (Table 2). These hydrogel models have been extensively studied and compared to 2D culture and in vivo conditions. Cells have been shown to have differing spatio-physical properties in 3D and conventional 2D culture conditions [116,117,118,119]. Hsieh and colleagues showed that cells cultured in 2D exhibit greater drug sensitivity than in vivo, and 3D cultures exhibit chemosensitivities comparable to solid tumors provided that they have similar cell density [120]. Expanding the cellular environment from 2D to 3D has also been shown to affect proliferation and metabolism [120,121,122]. Using 3D cultures has led to significant advancements in the understanding of cell migration strategies as well, since some of these could not be observed in 2D cultures [123,124,125]. For instance, fibroblasts have several different migration strategies that are utilized in different microenvironmental conditions, demonstrating cellular plasticity [125]. Such findings prompt questions about the mechano- and bio-sensing abilities of cells in relation to their environments. In this section, we will elaborate on the different methods utilized thus far to improve cell-microenvironment mimicry in the form of hydrogel models, the cellular response to such models in contrast with conventional cell-culture methods, and factors to be considered in crafting a hydrogel model.

#### 1.3.1. Mimicking Cellular Microenvironment Biomechanics

**Figure 3:**
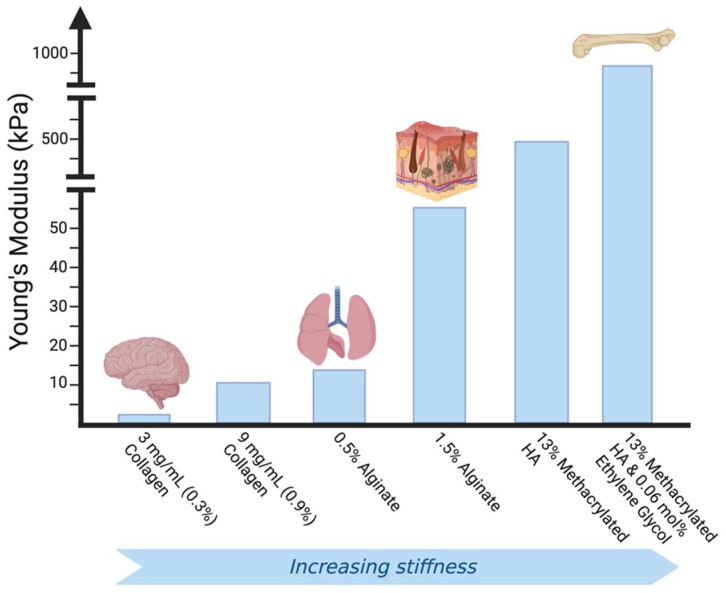
Young’s modulus of hydrogels. The reported Young’s modulus (kPa) values of various hydrogels are plotted with an image of the tissue that has similar Young’s modulus. It is crucial to choose a hydrogel model that corresponds to the in vivo mechanical properties of tissue that is relevant to the work. (Created with BioRender.com, accessed on 14 July 2022).

There have been many diverse approaches to capturing the complexity of the ECM and the biomechanical cues directed by it via in vitro hydrogel models. Tissue specificity is a crucial factor to consider in developing an ECM-mimetic hydrogel-based cell culture system, since the cells of each tissue produce and degrade matrix constituents, leading to variable ECM composition, bioactivity, and mechanics amongst tissues [129] (Figure 3). In the bone, the ECM is calcified to provide structural support to the tissue, while in tendons it is structured to provide tensile strength [130,131].

Most natural ECM mimics in vitro have been hydrogels composed of ECM components such as collagen, fibronectin, or hyaluronic acid. Such hydrogels have been significant in studying cellular behavior in 3D environments as well as cell response to controlled mechanical properties such as stiffness, elasticity, and rigidity [63,132,133,134,135,136,137,138,139]. Photo-crosslinking, chemical crosslinking, changing fiber density, and the development of ‘smart’ hydrogels that are responsive to external stimuli (e.g., changes in pH, temperature, and light) have all been used to fine-tune hydrogels to mimic the cellular microenvironment mechanically [140,141,142,143,144,145,146,147,148,149,150,151]. For example, photo-crosslinking of gelatin methacryloyl (GelMa) hydrogels alters hydrogel stiffness based on the light intensity, exposure time, and concentration of photo-initiator used in photo-crosslinking [143]. Nanocomposite smart hydrogels have been produced to change volume, Young’s modulus, and breaking strength based on applied chemical and physical stimuli [148,150].

#### 1.3.2. Recapitulating Cellular Microenvironment Heterogeneity

While the above-discussed methods are useful to study the effects of semi-isolated mechanical conditions on cells, they often disregard the biological complexity of the ECM. In vivo ECMs can also serve as a medium through which cells communicate with one another via secreted growth and signaling factors in vitro. Several protocols have been developed to decellularize ECM isolated from animal or human tissue, or to harvest ECM secreted by fibroblasts in vitro [152,153,154,155]. To better capture the biological complexity of the ECM, hydrogel models have been developed to incorporate fibroblasts, growth factors, and more diverse components of the ECMs specific to the cell type being studied. Incorporating fibroblasts in a co-culturing system with the cell type of interest is a relatively newer approach, which allows for fine-tuning mechanical properties using a hydrogel model while adding biological complexity to the culture through the addition of the secreted matrix from fibroblast cells [156,157]. Lee et al. developed a 3D hydrogel system for co-culturing human liver-cancer cell spheroids with fibroblasts on a micropatterned fibrous scaffold, thereby modeling the three dimensional structure of tumors and the cross-talk between cancer cells and neighboring fibroblasts [156]. Several groups have also developed methods of controlled growth factor release into hydrogel-based cell cultures [158,159,160]. It is crucial to combine the biochemical components of the ECM with the mechanical properties to better mimic the cellular microenvironment in vitro.

## 2. Hydrogels and Their Applications

Hydrogels are defined as hydrophilic polymer networks that form a three-dimensional structure [161]. A critical property of hydrogels is their ability to swell with water without dissolving due to hydrophilic functional groups present on the polymers of the hydrogel. Their high water content makes them flexible and resemble soft tissue, implicating their use in biomedical studies [162]. There are many polymeric substances that can be classified as hydrogels, and they are generally composed of one or more natural or synthetic materials for use in a wide variety of applications. The three-dimensional structure of hydrogels is preserved despite swelling by chemical and/or physical crosslinks within the polymeric network. Changes in composition, protein concentration, and crosslinking density lead to changes in elasticity, polymer density, and biodegradation rate [163]. These and other tunable properties of hydrogels make them suitable for a wide range of studies on mechanical, chemical, and biological conditions in vitro as well as ideal candidates for use in in vivo clinical applications such as in drug delivery systems [161,162]. Here, we describe the major classifications of hydrogels based on their composition, the general properties of hydrogels, and current and potential experimental and clinical applications of hydrogels with a focus on pre-clinical cancer models.

### 2.1. Types of Hydrogels

There are several classification methods for hydrogels, such as those based on ionic charge, biodegradability, physical properties, crosslinking, and preparation. They are most commonly classified by their source polymer(s): naturally occurring biomaterial, synthetic bio-mimetic, or a hybrid of these two sources [163]. Synthetic materials are traditionally used extensively due to low biodegradation rate, ease of manipulation, and greater control over biochemical interactions [149,164,165]. Natural biomaterials, however, are preferred due to their ability to biologically mimic the structural and biochemical properties of the cellular niche in vitro, and are responsive to cellular activities in terms of biochemical reactivity and degradability [164]. Numerous studies have presented methods for forming hybrid hydrogels that possess properties of both natural and synthetic biomaterials that are mechanically and biochemically responsive and tunable by the surrounding environment [165,166].

#### 2.1.1. Natural Hydrogels

Natural hydrogels are composed of naturally occurring polysaccharides and proteins such as collagen, Matrigel^®^, hyaluronan, gelatin, and their derivatives, including alginate and chitosan [167,168]. While most studies cite hydrogel formation from one ECM protein, there are several that utilized hydrogels composed of several proteins [167,168]. Either protein concentration or crosslinking density is altered in the hydrogels in order to change the Young’s modulus of the gels [167,168]. However, these are not finely tunable or as well-understood as the mechanical properties of synthetic hydrogels [169]. In contrast, the biocompatibility of natural hydrogels and their responsiveness to cellular degradation add an important dimension to biological studies [170].

**Figure 4:**
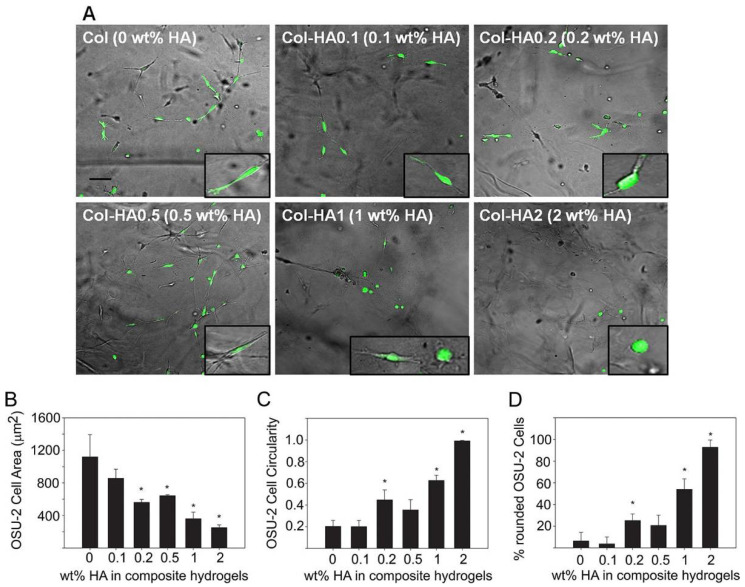
Glioblastoma cell morphologies and migration when cultured on different substrates. OSU-2 glioma cells were cultured on collagen I and III and HA composite hydrogels with different concentrations of HA. Cell morphologies shown in (**A**) were quantified via cell area (**B**), circularity (**C**), and roundness (**D**). Increased HA content led to lower cell area but increased cell circularity and roundness. The scale bar in (**A**) indicates 100 µm, * in (**B**–**D**) represents a *p*-value < 0.05 compared to 0% HA condition. Figure reprinted/adapted with permission from Ref. [171]. Copyright 2013, American Chemical Society.

Many studies incorporate several proteins into biomaterial hydrogels to improve hydrogel stability and similarity to the in vivo cellular microenvironment. Comparing cellular properties in vitro in composite hydrogels versus single-material hydrogels can give more specific information on how cell mechano-sensitivity is influenced by specific cell-ECM protein interactions. A useful representative investigation of the insight obtained by studies undertaken with composite hydrogels is a study by Rao et al. [171] (Figure 4). They mixed collagens I and III with hyaluronic acid (HA), collagen IV with HA, and used collagen (I and III) as a standalone in hydrogel formation to determine modulus values of the different hydrogels and observe changes in cell morphology, spreading, and migration. Composite hydrogels had much higher elastic modulus values than the collagen hydrogel, and increased HA content correlated to increased modulus values and greater cell spreading and migration [171]. Interestingly, cell morphology differed by the type of collagen used in the hydrogels: cancer cells cultured in collagen IV had a rounder cell shape, while those in collagen I and III were spindle shaped [171].

Another common composite hydrogel used in several cell mechanics studies is Matrigel^®^, a mixture of ECM proteins secreted from mouse sarcoma cells, which is commonly used for spheroid formation in 3D cultures [172]. Several mechanics studies use Matrigel^®^ for the formation of spheroids before seeding onto other types of hydrogels or for studies of cell invasion. For instance, Grundy et al. reported that primary GBM cells that were insensitive to rigidity were more invasive in spheroid cultures in Matrigel^®^ as opposed to rigidity sensitive cells [173].

Hydrogels using natural biomaterials are also advantageous, since they allow for the design of an in vitro extracellular environment that can biologically mimic the in vivo ECM and various cellular conditions. Natural hydrogels have been used for tissue engineering and regenerative medicine because they form structures similar to tissue ECM due to the natural proteins and polysaccharides that they are composed of [168]. While the biological similarity is closer to in vivo when natural biomaterials are used in hydrogel preparation, the mechanical properties are not adjustable or robust compared to those of synthetic hydrogels. Additionally, natural hydrogels can lead to great variation from batch to batch due to the components being sourced naturally [168,174]. Recently, however, ‘smart’ hydrogels, which are robust, have localized mechanical properties, and are responsive to changes in pH, temperature, and light have been developed from natural materials [148]. Smart hydrogels composed of natural materials have many possible applications in tissue engineering, regenerative medicine, and stem cell and cancer research.

#### 2.1.2. Synthetic Hydrogels

Synthetic hydrogels are composed of synthetic materials such as polyethylene glycol (PEG), poly(vinyl alcohol), and poly-2-hydroxy ethyl methacrylate [175,176]. They offer advantages to natural hydrogels in the sense that they offer greater control over gel mechanical properties, have higher capacity for water absorption, do not degrade as rapidly as biomaterial hydrogels, and have great reproducibility [177]. For instance, PEG-based hydrogels are useful in mechanics studies due to stiffness tunability and ability to support long culture periods (several weeks) [178]. Synthetic hydrogels have more utility in studies focused on the effect of isolated mechanical properties on cell behavior without the additional influences of interactions between cells and biochemically active biomaterials [179]. Biomimetic polymers have also been synthesized which may contain a similar component to a natural material, such as a specific amino-acid sequence, which can add biological activity such as degradability or biochemical signaling to the gel in a more controlled environment than if a natural biomaterial were used [180]. Therefore, synthetic hydrogels are extremely customizable and can be synthesized for specific medical or research applications.

Synthetic hydrogels have been used to study the mechanics of cancer, as reported in several previous publications. For instance, 2D “films” of poly-methylphenyl siloxane with increasing stiffness values resulted in increased cell spreading and migration of glioblastoma cells compared to more compliant films [181]. In developing an in vitro drug-screening platform for cancer, synthetic HA derivatives, HA-aldehyde and HA-hydrazide, were crosslinked and formed into a hydrogel where cells were able to form clustered structures similar to tumors, and had greater drug resistance than in 2D cultures [182]. PEG hydrogels are often used for hydrogel preparation due to their tunable properties and high biocompatibility compared to other synthetic materials. They were used in mechanical studies of lung adenocarcinoma where matrix stiffness alterations resulted in changes in cellular morphology [183].

While there are several advantages to using synthetic hydrogels, they are limiting in their ability to mimic the complexity of native ECM. This highlights the importance of efforts to add biochemical reactivity to synthetic hydrogels for in vitro studies that are aiming to mimic cellular microenvironments [184]. For instance, Lutolf et al. engineered synthetic hydrogels that were degradable by matrix metalloproteinases through crosslinking of synthetic substrates into the hydrogels [164]. Smart synthetic hydrogels have also been studied to engineer stimuli-responsive synthetic biomaterials that change when faced with altered temperature, pH, light, and other stimuli [185].

#### 2.1.3. Hybrid Hydrogels

Many hydrogels are composed of synthetic materials that are mixed, conjugated, or coated with biomaterials to provide researchers with insight into the controlled mechanical response and specific cell-protein interactions while maintaining the ability to adjust mechanical properties, and keep a low rate of degradation [186]. In general, a limiting property of hydrogels is their low stiffness and rigidity, which is the opposite of in vivo tissue properties. Hybrid hydrogels have been of interest in studies to improve both the stiffness and rigidity of hydrogels [177,187].

Many types of hybrid hydrogels have been developed for cancer mechanics studies. Fibronectin-coated PA substrates were used to study the invasiveness of different human glioma cell lines with a focus on cell structure, migration, and proliferation [188]. Cells were rounder and had lower migration and proliferation rates in ECM substrates with lower rigidity. GBM migration patterns in the brain white matter tract were mimicked in vitro with electrospun alignment of nanofibers in a mixture of gelatin, poly-ethersulfone, poly-dimethylsiloxane, HA, and collagen in order to study migration patterns of GBM cells, and the addition of HA was seen to have a converse effect on migration [189]. The addition of HA to gelatin and PEG composite hydrogels resulted in dose-dependent glioma malignancy marker expression changes and cell clustering [190]. Another study combined PA hydrogels with HA and either laminin or collagen I and found that collagen and laminin presence was correlated to mechanical response to substrate stiffness [134].

### 2.2. General Properties of Hydrogels

Hydrogels are defined by their hydrophilicity, which allows them to store water and swell without dissolving [191]. The bio-responsive properties of hydrogels, such as biochemical activity and degradability, allow for culture conditions to be dynamic and receptive to cellular cues. Conversely, since hydrogels are tunable in mechanical properties such as elasticity, compliance, and stiffness, it is possible to study the responses of cells to microenvironmental mechanics. While synthetic hydrogels are easier to adjust mechanically, natural hydrogels are more bioresponsive. In crafting a study, these are important properties to keep in mind, as they introduce variables to whichever system is being studied.

Two important descriptors of hydrogels that determine many physical properties are ionization degree and crosslink density. Crosslinks in a hydrogel are either chemical or physical, and can be introduced to a gel by methods such as irradiation, sulfur vulcanization, or chemical reactions aided by temperature and pressure [144]. Swelling and elastic modulus values are determined by cross-linking degree and charge densities or ionic strength of the polymers in the hydrogel [192]. Greater concentration of cross-linked polymers and the number of ionic groups cause higher elastic modulus values and greater swelling capacity [193]. Similarly, the distribution of proteins or polymers and cross-links in hydrogels, which are generally non-homogenous, are affected by cross-link density and degree of ionization [194,195].

There are numerous properties of hydrogels that can be influenced by internal factors, e.g., composition, protein concentration, and polymer modifications, and by external factors, e.g., UV radiation and temperature [161]. Chemical and physical reproducibility of hydrogels depends on controlled conditions and utility in research can be greatly enhanced by proper knowledge of hydrogel properties and subsequent unique modifications of the scaffold to better mimic whichever system is being studied [196].

### 2.3. Research Applications of Hydrogels

Research applications of hydrogels are varied in vitro. They are often used as scaffolds in 3D cultures for the study of cellular physiology and characteristics, as they interact with tissue-mimetic hydrogels. While 2D culturing methods have provided valuable information on cellular characteristics, recent studies have undertaken a shift toward preference for 3D-culturing methods for more advanced and sensitive studies that imitate native tissues and cells more closely [126,197]. These 3D platforms provide more physiologically relevant information on cell-environment interactions biochemically and mechanically, including: morphology, cell and environmental stiffness, motility, and signaling [117]. Some 3D platforms that have been used for cell culture include microporous and nanofibrous scaffolds encapsulating cells. However, these either have pore sizes that are too large, which negate the 3D structure and act as a 2D scaffold, or are too weak for mechanical studies [198,199]. Hydrogels are useful as mechanically and biochemically tunable matrices for 3D cultures, which simulate soft tissue structure and have potential for translation and clinical applications [184]. For instance, Jiang et al. found that the formation of a hybrid hydrogel with ultralong hydroxyapatite nanowires and sodium alginate allows for improved mechanical properties of the hydrogels and enhanced biocompatibility for in vitro studies [200].

Drug screening or efficacy assays are prone to showing promising effects in vitro, only to fail or be substantially less effective in animal models and clinical trials [201]. In the search for in vitro cell-culture systems that can provide more accurate and relevant results, 3D scaffolds such as hydrogels have been gaining attention, in part due to their potential to mimic the ECM and inhibit drug delivery [119]. Huber et al. compared the response of 2D cultures of non-small cell lung cancer cultured to 3D microtissues of the same cell line to various drugs and found that drug efficacy was significantly different between the models [127]. Singh et al. developed a hydrogel microarray assay to generate uniform microtumors and subsequently study tumor response to epidermal growth factor (EGF) and cetuximab treatments [202]. Such systems increase the chances of success for translation because they enable studies of treatment response more closely aligned to clinical response.

### 2.4. Clinical Applications of Hydrogels

A clinical approach to hydrogel scaffolds is found tissue engineering applications, which have gained traction recently as potential solutions to donor shortage problems for tissue or organ transplantations [203,204]. These approaches generally combine cells from a donor with a hydrogel scaffold that is prepared to mimic the extracellular matrix of the tissue or organ being engineered [203]. Hydrogels are suitable tissue-engineering applications due to their ability to uptake water, to encapsulate cells, and to be bio-reactive [8]. Various hydrogels have been used as bio-ink for 3D organ or tissue printing applications in which tissues are built with direct deposition of cells with the bio-ink [205]. Studies have shown applicability of alginate, collagen, and various composite hydrogels for tissue printing methods [206,207,208]. For instance, natural protein and polysaccharide hydrogels have been used for articular cartilage-tissue engineering applications to promote cartilage regeneration [209]. Latifi and colleagues demonstrated the potential of an injectable hybrid hydrogel (collagen I and III) to be applied in soft-tissue engineering, specifically human vocal fold engineering [210]. A long-standing problem of the tissue engineering field is the need for in vitro tissue vascularization to be able to transplant or implant larger portions of tissue into patients. A couple of groups have recently made strides in promoting in vitro vascularization of hydrogel constructs of bone [211] and soft tissue [212].

Injectable hydrogels have been studied for drug delivery and wound healing or dressing applications [213]. Hydrogels have been applied in drug delivery applications due to their ability to give control over the time and/or site of drug delivery for enhanced treatment [214,215]. The biocompatibility, similarity to native tissues, and high-water content all contribute to great applicability of hydrogels for controlled drug release and delivery [216]. Naturally derived injectable hydrogels for the controlled delivery of small molecules to the central nervous system have also been extensively studied. Wang et al. have shown that an injectable hyaluronan-methylcellulose hydrogel enhanced delivery of growth factors [217]. Further, the great tunability of hydrogels has led to development of thermosensitive, pH-sensitive, and temperature-sensitive hydrogels that can be used for drug delivery in distinct biological environments [218,219,220]. Similarly, temperature-sensitive hydrogels have been developed for various wound-healing applications. A PEG-PLGA-PEG composite hydrogel has been developed for delivery of a growth factor linked to tissue repair for diabetic wound healing [221].

Tissue regeneration is another field in which hydrogels are being investigated and have shown to be promising for translation to the clinic due to their biocompatibility and ability to be fine-tuned or adapted for specific applications. For instance, Zheng et al. developed a polyacrylic, acid-alginate-demineralized, bone matrix hybrid double-network hydrogel, which was shown to promote vascular endothelial growth factor (VEGF) synthesis and basic fibroblast growth factor (bFGF) and alkaline phosphatase activity of MG63 osteosarcoma cells to enhance bone regeneration [211]. Stem-cell therapy is a promising solution for injuries that require tissue regeneration, but it is limiting since uncontrolled differentiation can lead to the presence of unnecessary cells at the site of injury and lead to stem cell metastasis and tumorigenesis [43,60,129]. Application of hydrogels can improve stem cell therapy by introducing stem cells and differentiation factors to the injury in a site-specific manner [5]. In a study of spinal cord injury, Mothe et al. investigated a hydrogel-integrated stem cell therapy by encapsulating neural stem cells and differentiation factors in a hyaluronan-based hybrid hydrogel and found that treatment enhanced graft survival, increased oligodendrocytic differentiation, and reduced cavitation in the injury site in rats [222].

Contact lenses are an example of the clinical application of hydrogels [223,224]. While hard contact lenses are composed of hydrophobic materials, soft contact lenses are hydrogel-based [225]. Owing to the wide variety in hydrogel-forming substances, attempts are continually being made to improve the physical and chemical properties of contact lenses [225]. Hydrogels have also been used clinically in dermatology applications such as wound healing and skin regeneration [226,227].

## 3. Cancer and the Tumor Microenvironment

Biomechanical properties of the tumor microenvironment have been shown to be altered compared to the healthy state in many types of cancer to promote processes crucial to tumorigenesis, including cellular proliferation and migration. Since changes in extracellular mechanical properties can induce structural reorganization, morphological changes, and altered signaling, they can cause cancer cells to exhibit mechanical properties differently than healthy cells, e.g., stiffness [228]. This, in turn, can further promote invasive or metastatic phenotypes [229]. For instance, cancer cells also usually have a lower Young’s modulus compared to healthy cells of the same type, which can influence deformability and influence migratory ability [230]. Cancer cells also have a more robust ability to respond to ECM conditions, and can alter cytoplasm viscoelasticity in response to increased ECM stiffness and collagen I deposition [231].

Breast cancer is a context in which mechanical properties of tissue and cells have been well described, and many biomechanical contributions to carcinogenesis and metastasis have been identified [96]. Many of the studies involving cell response to mechanical stimuli have been conducted with aggressive breast-cancer cells, especially the MDA-MB-231 cell line, due to their robust response to changes in extracellular mechanics. Clinically, tissue stiffness has served as an indicator of breast tumors and risk of breast cancer [232,233], and the biomolecular consequences of this phenotype has been studied rigorously in many types of 3D hydrogel models in the laboratory, especially in the last decade. Various mechanical stimuli can cause cellular stress and lead to carcinogenesis or increased invasiveness of cancer cells. For instance, several mechanotransduction pathways are linked to carcinogenesis and invasiveness and upregulated in cancer [95,100,234,235]. Application of mechanical load was shown to regulate breast-cancer cell proliferation independent of matrix deformations or stiffness [234]. Mechanical stretch, ECM stiffness, and fluid shear-stress all led to more invasive phenotypes of breast-cancer cells. At the level of response to treatment, ECM stiffness has been characterized to contribute to chemoresistance of breast-cancer cells to doxorubicin [235]. Therefore, a potential therapeutic approach may be to introduce proteinases or drugs that reduce ECM stiffness by degrading certain components to reduce the number of treatment-resistant cells. Lastly, certain microenvironmental properties have been correlated to improved prognosis in breast cancer and can be used to identify diagnostic and prognostic signatures. For instance, the tumor-associated collagen signature consisting of aligned collagen fibers in biopsied tissues from breast-cancer patients has been identified as a prognostic signature for survival [236].

The positive contributions that studying biomechanics has made to our overall understanding of breast cancer highlight the importance of incorporating a biomechanics-based approach to cancer biology in vitro studies. Inspired by the benefits of biomechanics studies on our understanding of breast cancer, we will present the current research in glioblastoma (GBM), which is a disease that has not been well-defined biomechanically. We believe that a better understanding of the biomechanical properties of GBM and its microenvironment can produce translatable results that may contribute to the development of diagnostic and therapeutic approaches to improve the prognosis of this invasive cancer.

### 3.1. Glioblastoma and the Tumor Microenvironment

Biomechanical and biophysical studies can help to glean valuable insight into a wide variety of diseases, since biomechanics is an integral part of cell proliferative, migration, and survival signaling, all of which are crucial to carcinogenesis and tumorigenesis. GBM is the most common and lethal form of primary brain cancer in adults [237], but there are only two clinically approved chemotherapies targeting it. It is an example of a disease that has not been traditionally studied in terms of biomechanics until approximately the last ten years but is one that may greatly benefit from such studies. Here, we will present an overview of the biomechanical properties known about GBM, the methods and contributions of available 3D culture and mechanotransduction studies of GBM, and the potential translational impact of those studies on the clinic and patient survival.

#### 3.1.1. The Blood-Brain Barrier

The blood-brain barrier (BBB) is also an essential part of the brain microenvironment that is altered in the cancer state. The BBB is formed by vascular endothelial cells lining microvessels in the brain and is essential in regulating brain extracellular conditions to ensure neuronal signaling [238]. The endothelial cells of the BBB limit transcellular and paracellular transit into, and thereby protect, the brain by regulating permeability through tight junctions, adherens junctions, charged moieties, pericytes, etc. [239,240]. In tumor microvessels, however, loss of claudin-1 and claudin-3 and down-regulation of claudin-5 was observed, which correlated to increased permeability [241,242].

#### 3.1.2. Extracellular Matrix of the Brain

**Figure 5:**
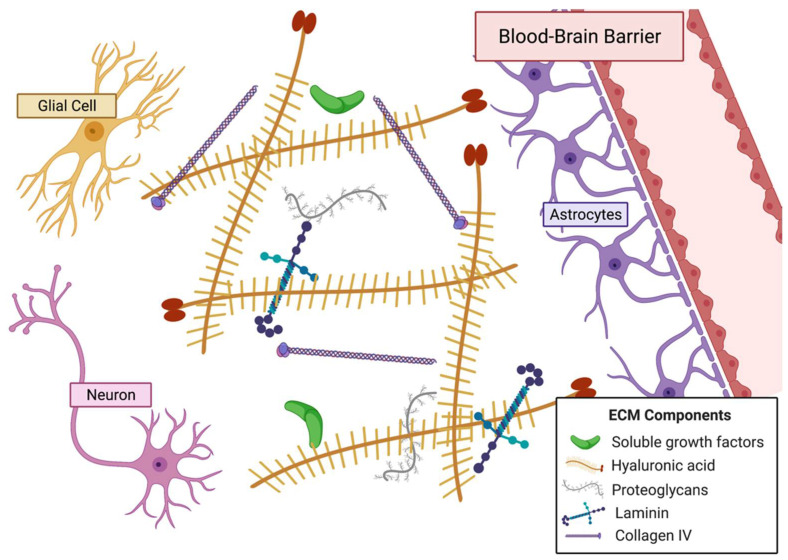
Extracellular matrix (ECM) of the brain. The ECM of the brain has a unique composition, including hyaluronic acid, collagen IV, and other ECM components along with glial cells, neurons, and astrocytes. The blood-brain barrier (BBB) is a neurovascular unit composed of vascular endothelial cells with surface charge modifications, tight junction proteins, pericytes, astrocytes, and other components. The BBB is selectively permeable and can block solutes in the systemic blood from entering the environment of the central nervous system (Created with BioRender.com, accessed on 14 July 2022).

The ECM of the brain (Figure 5) is altered when tumorigenesis occurs. Several ECM components, including HA, tenascin-C, and vitronectin, are upregulated in the tumor microenvironment [243]. Studies show that proteins of the tumor niche also tend to be different than healthy brain tissue and that tumor invasion alters ECM composition [244]. Basement membrane components such as laminin, fibronectin, and collagen type IV are more highly secreted by glioma cells and, in turn, alter composition of the local ECM [245,246]. Tumor-associated mesenchymal stem-like cells induce HA synthase 2 activity and lead to greater HA abundance in the tumor niche [247]. In addition to a distinct microenvironment, the GBM tumor has a hypoxic and necrotic core that aids in the cancer cell-induced blood vessel formation by increasing expression of pro-angiogenic VEGF, VEGFR2, and angiopoietin 2, which results in the disorganized network of blood vessels observed in GBM [243,248,249,250].

Many cellular properties are altered in the cancer state. For instance, cell proliferation, migration, and deformability is increased in the GBM state when compared to healthy cells [67,138,251,252,253,254]. The impact of the extracellular niche on these cellular properties and the biology behind the changes have been elucidated by various in vitro and in vivo studies focusing on mechanical cell-ECM interactions [17,101,255,256,257].

#### 3.1.3. Overview of Microenvironment and Biomechanics of Glioblastoma

A large body of research has been dedicated to studying the differences between the microenvironments, or niche, of tumor and healthy cells. These include studies on overall tissue stiffness, ECM composition, cellular signaling, and the presence/activation of mechanotransducers. There is a general consensus that while cancer cells are less stiff and more deformable than healthy cells [28,105,106,255,256], tumor tissue tends to be stiffer by variable magnitude compared to non-tumor tissues, a trend that has been shown in thyroid, breast, prostate, bladder, and kidney tissues [107,108,109]. The tumor niche is also characterized by altered ECM composition, which may lead to increased invasiveness and metastatic properties of cancer cells [257]. Characterization of the ECM, both biologically and mechanically, in GBM has not reached the depth of understanding that has been achieved for several other types of cancer, such as prostate and breast cancer. However, there are certain differences that have been noted between healthy and cancerous tissue microenvironments, which can lay the groundwork for future studies of GBM microenvironment and mechanics.

##### Biomechanics of the Glioblastoma Extracellular Matrix 

The mechanical properties of the tumor niche in GBM are different than those of the healthy brain ECM due to the altered composition, protein-protein, cell-protein, and cell-cell interactions. Even within the GBM tumor, there are distinct mechanical regions for necrotic and non-necrotic portions [250]. In general, GBM tissue has been found to be stiffer than healthy ECM, with increasing stiffness generally correlating to increased malignancy [109,147]. From a set of human brain biopsies, one study showed that increasing malignancy of tumors gave higher Young’s modulus values, with primary GBMs exhibiting stiffness values varying from 70 to 13,500 Pa [258]. Stewart et al. showed that brain tumors had elastic moduli ranging from 170 to 16,060 Pa using a custom-built indenter [259]. Another study correlated increased ECM stiffness to decreased survival of human patients [260]. Altered stiffness results in changed mechanical cues that are relayed to the cell, which then impact cellular gene expression so significantly that the overall behavior of the cell can be drastically changed [9] (Table 1). Several studies have been published that note differences in cellular morphology, deformability, motility, proliferation, and signaling in response to changes in environmental stiffness [104,134].

##### Modification of the Extracellular Matrix in the Brain by Glioblastoma Cells

The tumor also modifies the local ECM through protein degradation. Some of the most studied cell-secreted proteins to be up-regulated in the cancer state are matrix metalloproteinases (MMPs), which are proteases that remodel the ECM by degrading certain component proteins [261]. The hypoxic core of GBMs has been shown to be a significant contributor to the increased MMP activity [262]. Various MMPs that are up-regulated in GBMs have also been shown to aid in glioma cell invasion [263]. Plasminogen activation by the urokinase pathway, which includes urokinase (uPA), urokinase receptor (uPAR), and plasminogen, is also prominent in GBMs. The urokinase pathway aids in ECM degradation and remodeling by converting plasminogen into active plasmin, which is a serine kinase that degrades certain ECM proteins, and activates MMP-2 and MMP-9 [264]. MMP-2 is a type IV collagenase that has been implicated in invasion and metastasis of GBMs [263]. Lastly, various cathepsins, which are lysosomal cysteine proteases that can be secreted into the ECM, are also up-regulated in GBMs and have been linked to tumorigenesis and invasion [265,266,267].

#### 3.1.4. Glioblastoma Migration, Invasion, and Mechanotransduction

Some hallmarks of GBM are enhanced cellular migration and aggressive invasiveness [268]. These properties are achieved through complex mechano-chemical signaling mechanisms that enable crosstalk between the tumor cells and the tumor microenvironment. The proteins that sense and translate mechanical cues from the ECM or microenvironment and relay them to the cell are called mechanotransducers [54]. Interestingly, one study found that keeping the mechanotransducer RhoA GTPase constitutively active in vitro in U87 cells caused similar toxic responses in 3D environments with varying stiffness, indicating the importance of mechanotransduction in cell response to environmental conditions [133]. Another study found that by altering mechanotransducers in GBM tumor-initiating cells, they were able to alter cell motility and invasion in 3D cultures [67]. Knock-down of CD44, a transmembrane glycoprotein receptor for HA and other ECM components, resulted in decreased microtubule and vimentin expression, hampered migration, and decreased nuclear stiffness compared to control cells [104]. Integrins are well-studied mechanotransducers that are linked to malignancy and are primary communicators in cell-ECM adhesion and signal transduction [134,245,263]. Clinical nuclear medicine studies have evaluated the role of various positron emission tomography (PET) radiotracers (e.g., [^18^F]Galacto-RGD [269], [^68^Ga]PRGD2 [270], and [^18^F]FPPRGD2 [271]) to enable molecular imaging of integrin a_v_b_3_, a member a class of adhesion molecules that mediate cell–cell and cell-ECM interactions, and which plays an important role in cancer metastasis and angiogenesis [272]. These studies reveal the importance of understanding how mechanotransducers process mechanical cues from the ECM to influence cancer-cell properties [93,94].

**Figure 6:**
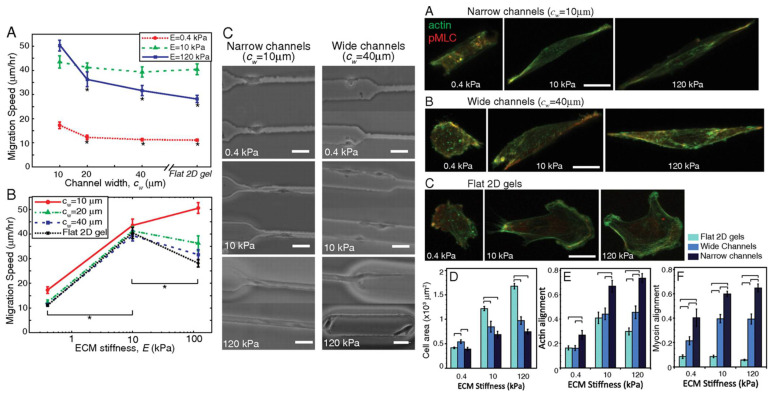
Cell migration, cell area, actin alignment, and myosin alignment when exposed to different channel widths and hydrogel stiffnesses. (**A**–**C**, **left panel**) Migration speed was quantified for cells cultured in narrow or wide channels with varying stiffness. With increased stiffness but decreased channel width, cell migration speed was higher. * *p* < 0.05. (**A**–**D**, **right panel**) Cell area, actin alignment, and myosin alignment were quantified from cells imaged after culture in wide or narrow channels with varying stiffness. Cells cultured in stiffer conditions with narrow channels exhibited increased actin and myosin alignment but lower cell spreading than those in narrow channels. Figure reprinted/adapted with permission from Ref. [102]. Copyright 2012, National Academy of Sciences.

Cellular structure and cytoskeletal alterations are under the direct influence of ECM mechanical cues and cellular mechanotransducers [93]. Pathak and Kumar found that culturing cells in narrow versus wide channels of various extracellular stiffnesses led to altered cell morphology, migration, and myosin alignment (Figure 6), underlining the importance of extracellular culture conditions in determining cell behavior. Various integrins serve to form attachments to proteins of the ECM. Once an integrin is bound to an extracellular ligand, focal adhesion clusters form at the surface of the integrin receptor within the cell, which link to the cytoskeleton and function in cell motility by recruiting proteases for ECM degradation and activating signaling pathways that induce cytoskeletal rearrangement [55]. Cytoskeletal rearrangement as orchestrated by cell-ECM interactions are vital to increasing cell deformability and, in turn, enhancing migration.

#### 3.1.5. In Vitro Studies of Mechanotransduction in Glioblastoma

Studies of GBM cell-ECM interactions and mechanics rely on in vitro matrix-mimetics that are composed of either biologically occurring proteins or synthetic materials. In 2D cultures, this is accomplished by coating glass or polystyrene with bio- or synthetic materials and culturing cells on top of the coating. In 3D cultures, hydrogels, defined as crosslinked polymer networks that can retain water, are composed of one or several synthetic or biomaterials to mimic the cell microenvironment. Changes in composition, component concentration, and crosslinking density lead to changes in rheological properties such as elasticity and stiffness [177]. Cell migration, morphology, cytoskeletal structure, invasiveness, and signaling are some of the properties characterized in gels of varying stiffness.

It is common practice in the study of GBM mechanics to use various naturally occurring proteins of the brain ECM to construct hydrogels in which cells are seeded. While most studies cite hydrogel formation from one ECM protein, there are several that utilize hydrogels mixed with several proteins, which are discussed in the next sections. Either protein concentration or crosslinking density is altered in the hydrogels to change Young’s modulus of the gels and study the effect of elasticity or stiffness in the microenvironment on the cells. The use of biomaterials in hydrogels offers the additional variable of gel degradation by cells or over time in incubation, which adds an important dimension to understanding tumor-cell function in remodeling the ECM.

#### 3.1.6. Hydrogel Culture Methods

##### Collagen

Despite collagen type I not being an abundant protein in the GBM and healthy brain ECM, it is often used in mechanics studies of these cells, due to its ability to easily form a gel. Even though collagen IV is upregulated and more abundant in the GBM microenvironment, there is a limited amount of studies in which collagen IV is used for hydrogel formation to investigate GBM mechanics [171]. There are commercially available pre-coated glass slides that make mechanics studies using collagen I simpler. In studies where glioma cells were seeded onto collagen I-coated substrates (glass or polystyrene), there was a trend of cells spreading more extensively in stiffer ECMs [76,273]. One group observed a nearly linear 80 μm extension called an “invadopodia” that stretched from one cell to another, which they hypothesized was a significant factor in cell signaling [273]. The drawback of studies using collagen-coated glass or polystyrene is that it forms a 2D culture, which does not accurately mimic the 3D environment that cells are exposed to in vivo. It was shown that GBM cells exhibited chemoresistance to sunitinib, a kinase inhibitor, in vitro in a 3D collagen-based environment, but not on plastic or collagen-coated surfaces [136]. Additionally, 2D cell migration does not require MMP activity, while 3D culture does, so MMP expression can be studied extensively in relation to mechanics only in 3D culture [274]. For greater insight into in vivo characteristics, it is more useful to employ 3D rather than 2D cultures.

Collagen fiber density or concentration is an important factor to consider in experimental design. A study confirmed that varying the collagen I concentration resulted in varying elastic modulus of hydrogels, but that the difference is not significant with collagen concentrations from 1.5 to 2.5 mg/mL [275]. They also found that a higher concentration of collagen inhibited growth of the GBM in vitro. Decreasing collagen gel stiffness was shown to increase migration distance and velocity of GBM cells [124]. Another study investigated the effects of gelation temperature on collagen I gel pore size and how this impacted glioma invasion. Gelation temperature caused variations in pore size for hydrogels composed of 1 mg/mL collagen and was a significant factor in determining speed of invasion of glioma cells with smaller pore sizes (5-12 μm) hindering cell motility [276]. A study by Hwang et al. showed that actin filamentation of migrating cells in collagen gels is dynamic and undergoes rapid changes, and produces of stress fibers and lamellipodia [277]. This study also drew attention to the migratory patterns of glioma cells, which was shown to mostly be composed of double-nucleated cells that migrated in clusters and had extensive interactions, through actin filament extensions, to collagen in the surrounding gel and nearby cells [277]. Distinct cell shapes, in terms of elongation and roundness, have been observed in different collagen gels [278]. Collagen gels were also used to study the effect of the microenvironment on gene expression and the effects of depletion or overexpression of certain genes on cell ability to retain mechano-responsiveness [278,279,280].

Three-dimensional culturing methods are being modified with novel technologies to model more than just a homogenous tumor microenvironment; they are being used to form models of interacting systems. In a study by Chonan et al., the tumor niche including ECM and blood vessels was mimicked in a microfluidic device using collagen I and human umbilical vein endothelial cells (HUVECs) [281]. Further studies with collagens are required for a more thorough understanding of the effect of these proteins on GBM cells.

##### Hyaluronan

There is extensive research on the production of hyaluronan (HA)-based gels for studies of GBM mechanics. HA hydrogels have been used clinically as implants for neural regeneration and reduction of scar formation [282,283]. In in vitro studies, HA gels are generally functionalized with peptides or other ECM proteins, such as laminin, RGD (Arg-Gly-Asp peptides), poly-D-lysine, and poly-L-lysine, which allow for cell adhesion to the gel, since HA alone does not attach to the cells [283,284]. In a study with short Arg-Gly-Asp (RGD) peptides incorporated to HA gels, it was noted that glioma cells’ actin stress fiber assembly and cell spreading was greater in stiffer gels [147]. Various HA crosslinkers can also be used to increase gel stiffness. Divinyl sulfone, for example, was used to crosslink HA carboxyl groups to varying degrees when different concentrations were used [285]. Chitosan-HA scaffolds, when compared to 2D surfaces, increased invasiveness and chemotherapeutic resistance and were proposed as possible in vitro mimics of the tumor microenvironment for pre-clinical drug effectiveness studies [286].

##### Other Proteins

Proteins such as fibronectin, laminin, and gelatin on their own are not commonly used for hydrogel formation. Rather, they are more frequently used to coat glass or polystyrene for two-dimensional cell-protein interaction studies. In the formation of hydrogels, they are generally used in combination with synthetic materials. These were also discussed previously in the synthetic hydrogels section of this review.

Methacrylated gelatin (GelMA) has been used on its own as a scaffold for glioma cells in a study of gel biophysical properties and their effect on cell morphology, proliferation, motility, and gene expression. Pedron et al. observed that GelMA biophysical properties could be varied by the methacrylation degree and bulk density. Cell morphology, motility and expression of hypoxia markers (VEGF, MMP-2, MMP-9, and HIF-1) and the ECM protein fibronectin were affected by the environmental properties [287]. Ramamoorthi et al. used an alginate hydrogel with varying stiffness values and observed greater cell sensitivity to toxins in less stiff gels [133].

##### Composite Biomaterial Hydrogels

There is a paucity of research involving composite hydrogels in the study of GBM mechanics. Researchers developed a composite matrix of HA and collagen oligomers with the addition of Matrigel-coated microfibers with tunable stiffness by varying component protein concentrations [124]. While low stiffness correlated to lower migration velocity and distance, collagen source and concentration was shown to affect these parameters variably. They also reported that in the HA hydrogel, cells exhibited collective migration while in collagen, they relied on single-cell migration [124]. These studies outline some of the different cellular responses to each of the ECM components and serve to emphasize the importance of studying specific cell-ECM interactions when attempting to describe GBM mechanics.

##### Synthetic Hydrogels

There are several types of synthetic polymers that have been used in the study of GBM mechanics. In 2D culture, “poly-methylphenyl-siloxane film” with greater stiffness values resulted in increased cell spreading and migration compared to more compliant films [181]. Wan et al. developed nanotextured polydimethylsiloxane (PDMS) surfaces with aptamers overexpressing epidermal growth factor receptor (EGFR) to isolate human GBM cells from a mix with fibroblast cells [288]. PA hydrogels are commonly used in GBM mechanics studies. In a novel study of cell mechano-sensitivity to matrix confinement, cells that were seeded onto PA hydrogels in more narrow channels exhibited greater migration speeds when compared to cells seeded on PA hydrogels in wider channels or directly onto 2D surfaces. This effect was abrogated by the inhibition of non-muscle myosin II, implicating this protein as a mechanosensory [102]. In a study investigating patient-derived primary GBM cell sensitivity to gel stiffness or rigidity, cells were seeded onto PA gels with different rigidity measures and the migration rate was correlated to rigidity-sensitivity [173]. Human glioma cell lines U373 and U118 cultured on polyacrylamide gels of normal brain stiffness (1 kPa) and GBM tumor stiffness (12 kPa), had greater proliferation rates in the stiffer substrate [289]. Umesh et al. studied the effect of PA gel stiffness on expression of proteins related to the cell cycle and dependency on EGFR signaling in human GBM cells, and saw that increased stiffness caused increased expression and phosphorylation of EGFR and Akt. Conversely, loss of EGFR, Akt, or phosphoinositide 3-kinase (PI3K) function resulted in decreased stiffness-sensitivity of the cells [252].

## 4. Challenges and Future Directions

**Figure 7:**
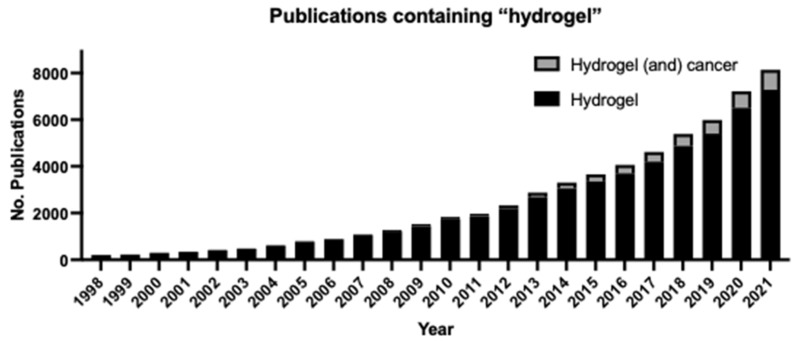
A bar chart showing the number of publications containing the key word “hydrogel” or (“hydrogel” AND “cancer”) from the years 1998 to 2021 found in the PubMed database.

We have endeavored to provide a thorough explanation of the various hydrogel-based methods that are employed for biomechanical studies and an overview of how studying cell-ECM interactions can lead to significant advancements in understanding the pathology of cancers, with a focus on GBM. It is abundantly clear from the state of the field that cellular response to biomechanical cues is a key player in maintaining health and homeostasis. To incorporate biomechanical effects into our current understanding of cell biology, hydrogel-based three-dimensional models of cell culture must become more widely used. The number of publications mentioning hydrogels has increased exponentially over the past 25 years (Figure 7). Furthermore, publications mentioning both hydrogels and cancer are making up a greater proportion of the hydrogel-related manuscripts published each year since 2012, indicating a growing interest in the development and use of hydrogels in the study of cancer. However, a major drawback of these approaches is that any one hydrogel model is not universally applicable due to the unique ECM composition and mechanical properties of the suite of cellular states that may be studied. Additionally, differences in hydrogel composition (and lot-to-lot variability of commercially available hydrogels), cell passage number, cell seeding density, hydrogel crosslinking density, time between cell seeding and microscopy, and other factors make it difficult to obtain consistent results [290,291]. It is worthy of note that ECM composition still has not been characterized based on the abundance of each component and elastic modulus for many tissues and conditions. Protocols unique to the disease model (cell type, microenvironment, perfusion, etc.) need to be established to enable wider adoption of hydrogel-based cell culture studies as an improved biomimetic replacement for conventional two-dimensional cell culture.

The use of hydrogels as model systems for studying cell response to biomechanical stimuli has been discussed in this review, with a focus on GBM as a case study for what has been achieved versus what has yet to be understood in the field. Breast cancer is an exemplar of the impact that biomechanical studies can have on diagnostic, prognostic, and therapeutic approaches to a disease. Therefore, we believe that future studies to understand how cells respond and adjust to mechanical stimuli, and how these responses may be dysregulated in various pathologies, are of utmost importance to craft a more systematic understanding of diseases that have been difficult to treat and cure, such as GBM. An improved understanding of tissue and cellular mechanics would facilitate the development of mechanotherapies for regenerative rehabilitation [292,293,294,295]. Not only will such studies further our understanding of the disease, but they may also provide clues for how to take advantage of mechanical stimuli to treat them. Hydrogels are used in contact lenses and as vehicles for drug delivery; they also have the potential to be used as therapeutic agents in cases where mechanical cues such as stiffness or stress can influence therapeutic resistance in certain cancers. For example, hydrogels with low stiffness may be implanted after tumor removal in patients with invasive cancers to reduce the ability of cancer cells to migrate. Such innovative uses for hydrogels and applications of knowledge that can be gained by biomechanical studies would improve our understanding and treatment of cancer.

## Figures and Tables

**Table 2:** 2D versus 3D cultures [117,118,119,123,126,127,128].

2-Dimensional Culture		3-Dimensional Culture	
Advantages	Disadvantages	Advantages	Disadvantages
Simple	Does not mimic in vivo structure	More like in vivo structure	Expensive
Reproducible	Fewer interactions with environment	Niches are available	Time consuming
Inexpensive	Access to unlimited amount of nutrients from media	Access to nutrients is not unlimited, varies	Less reproducible
	Less diverse phenotype and polarity	Can form organs or spheroid clusters of cells	More complex and difficult to carry out
	Altered cell morphology	Allows study of cell-cell and cell-ECM interactions	Fewer interactions with environment

## Data Availability

Not applicable.

## Abbreviations

Arg-Gly-Asp peptides	RGD
basic fibroblast growth factor	bFGF
blood-brain barrier	BBB
C-X-C motif chemokine receptor	CXCR
epidermal growth factor	EGF
epidermal growth factor receptor	EGFR
extracellular matrix	ECM
G-protein coupled receptor 68	GPR68
gelatin methacryloyl	GelMa
glioblastoma	GBM
human umbilical vein endothelial cells	HUVECs
hyaluronic acid	HA
linker of nucleo- and cyto-skeleton	LINC
matrix metalloproteinase	MMP
Myocardin related transcription factor A	MRTF-A
Neurogenic locus notch homolog protein 1	NOTCH1
phosphoinositide 3-kinase	PI3K
physically interacting cell sequencing	PIC-seq
platelet endothelial cell adhesion molecule-1	PECAM-1
polydimethylsiloxane	PDMS
polyethylene glycol	PEG
positron emission tomography	PET
rho-associated, coiled-coil-containing protein kinase	ROCK
T-cell receptor	TCR
Testin LIM domain protein	TES
Three-dimensional	3D
Transforming growth factor beta 1	TGF b1
Transient Receptor Potential Cation Channel Subfamily M Member 7	TRPM7
Transient Receptor Potential Cation Channel Subfamily V Member 4	TRPV4
Two-dimensional	2D
urokinase	uPA
urokinase receptor	uPAR
vascular endothelial growth factor	VEGF
vascular endothelial growth factor receptor	VEGFR
Vasodilator stimulated phosphoprotein	VASP
von Willebrand factor—glycoprotein Ib complex	VWF-GPIb
Yes-associated protein	YAP
